# Micro‐Embolic Events and Their Clearing in the Brain. A Narrative Review

**DOI:** 10.1111/apha.70098

**Published:** 2025-09-10

**Authors:** Kevin Mol, Inge A. Mulder, Ed van Bavel

**Affiliations:** ^1^ Biomedical Engineering and Physics Amsterdam University Medical Center, University of Amsterdam Amsterdam the Netherlands; ^2^ Amsterdam Neuroscience Neurovascular Disorders Amsterdam the Netherlands; ^3^ Amsterdam Cardiovascular Sciences Microcirculation the Netherlands

**Keywords:** angiophagy, cerebral ischemia, extravasation, microcirculation, microplastics, stroke, thrombosis

## Abstract

**Background:**

The cerebral circulation is continuously challenged by intravascular micrometer‐sized particles that become trapped microvascular‐emboli. These particles may include micro‐thrombi, stiffened erythrocytes, and leukocytes, while also fat particles, air, and microplastics may cause microvascular embolism.

**Review Scope:**

In this narrative review, we discuss these embolization processes and their acute and chronic consequences. These relate to the local flow interruption as well as the direct interaction with the endothelium. In addition, we address the clearing processes, including local thrombolysis and extravasation, or angiophagy, of the emboli.

**Conclusion:**

A continuous balance exists between embolic events and their resolution under normal conditions. Increased micro‐embolic rates, as occur in e.g., atrial fibrillation, or decreased clearing, possibly related to endothelial cell dysfunction, disturb this balance. This could lead to continuing loss of capillaries, micro‐infarcts, and cognitive decline.

## 1 Introduction

**FIGURE 1:**
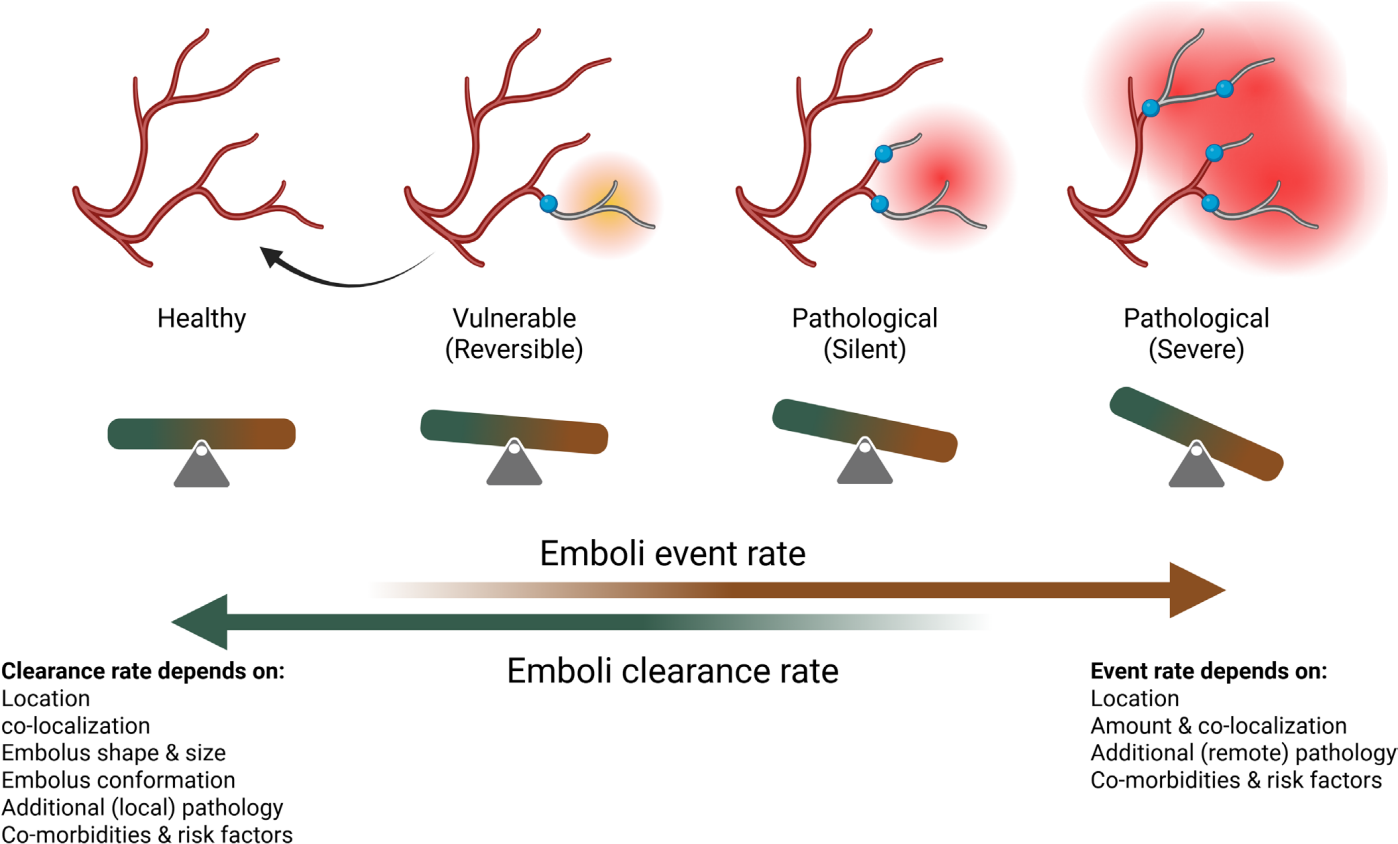
Sequence of events following micro‐embolization by a range of possible emboli. Depending on embolus type and size and endothelial health, lodging induces a range of processes at the seconds to hours timescale. These processes eventually will lead to either the development and growth of small infarcts or tissue recovery. *Created in BioRender* [2].

The microcirculation plays a crucial role in distributing oxygen and nutrients throughout the brain via an intricate network of small arteries, arterioles, and capillaries, followed by venules and veins. In mammals, capillaries typically measure around 5 μm in diameter, facilitating optimal gas and nutrient exchange while allowing the passage of erythrocytes, platelets, and leukocytes under normal physiological conditions. However, abnormalities can arise. Particles larger than capillary diameter can become trapped in the network, obstructing blood flow and oxygen delivery. Although embolism by a large, millimeter‐sized thrombus is relatively rare, its consequence—acute ischemic stroke—can be devastating [1]. Smaller thrombi embolize more distally, potentially with less severe outcomes or even remaining unnoticed (silent brain infarction). Yet their occurrence may be far more frequent. Quantitative data on this topic remain scarce, but substantial amounts of micro‐thrombi are likely to form in chronic conditions like atrial fibrillation (AF) and atherosclerosis, leading to increased embolism in the distal microcirculation, particularly in capillaries (Figure 1) [3, 4].

**FIGURE 2:**
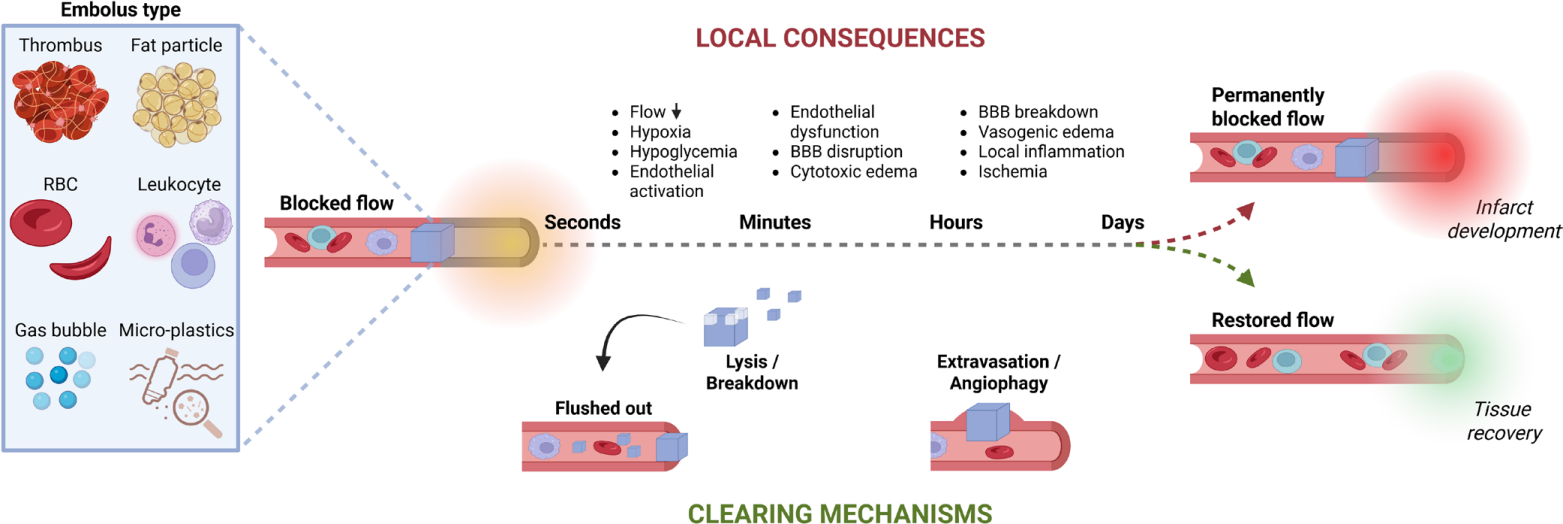
The cerebral microcirculation is continuously subjected to micro‐emboli. We suggest that under healthy conditions, the event rate is low enough to allow for clearance and local reperfusion. However, this balance is challenged by increased event rates or impaired clearance, both of which may result from cardiovascular health waning. Such a disbalance leads to progressive loss of capillary patency, culminating in silent or overt brain infarcts as well as continuous cognitive decline. *Created in BioRender* [5].

Beyond micro‐thrombi, arterial blood may contain other particles that contribute to microvascular embolisms, including rigid erythrocytes, activated leukocytes, circulating tumor cells, air emboli, and fat emboli from medical procedures (Figure 2). Moreover, growing concerns regarding the health risks associated with nano‐ and microplastics have sparked extensive research into their presence and toxicity in the human body. Microplastics, defined as exceeding 1 μm in diameter, have been detected across various organs and fluids, including blood and the brain [6, 7]. Limited quantitative studies found particles up to 20 μm in the circulation [8]. Due to their persistence and inability to degrade, these particles accumulate in tissues following microvascular entrapment. In the past decade, yearly increasing numbers of plastic particles in the liver and brain have been found in decedent human brains [9, 10].

Irrespective of their origin, capillary microvascular emboli (ME) need to be cleared in order to prevent continuous accumulation (Figure 1). For micro‐thrombi, degradation by plasmin seems to be an expected mechanism, but the sparse available data indicate that degradation and wash‐out rapidly become inefficient after the lodging of the clot [11, 12]. Plastic microparticles cannot be degraded, and capillary recanalization requires alternative processes that are based on active endothelial cell remodeling, including extravasation of the embolus. Remarkably little is known with regard to these processes. Accordingly, the purpose of this narrative review is to provide an overview of data and thoughts about microvascular obstruction and clearance.

Below, we first discuss the acute events that may cause microvascular embolisms, the nature of ME that can occur in chronic settings, and their consequences, from local tissue ischemia to long‐term organ damage. We then address capillary processing of ME and the current insight into the underlying cellular and molecular processes. We end by discussing the (im‐)balance between capillary embolic events and their resolution. While inflammatory responses and blood–brain barrier (BBB) disruption are heavily involved in the consequences of ME, we only briefly touch upon these processes.

## 2 Micro‐Embolic Events

Below we focus on embolic events in specifically the microcirculation, notably the capillaries with inclusion of arterioles and venules where relevant. Embolic events, notably thrombo‐embolism, may occur in any part of the vascular system, including large arteries and veins. Where relevant, we extrapolate from data on these large vessels.

### 2.1 Micro‐Thrombi

Micro‐thrombi are small, intravascular aggregates of platelets, fibrin, red blood cells, immune cells, and other coagulation components that occlude distal cerebral microvessels [13]. Due to their small size, it is challenging to detect micro‐thrombi by means of MRI or CT, while these embolisms remain predominantly clinically asymptomatic. However, several studies address the clinical relevance of micro‐thrombi as they can induce silent brain infarctions [14, 15, 16, 17], which occur with increased frequency during aging [18, 19]. Increased occurrence of micro‐thrombi and micro‐infarcts is also seen in chronic diseases like cerebral small vessel disease (CSVD) [20, 21], hypertension [22] and diabetes mellitus [23], where they are part of a detrimental loop of cause and consequence.

#### 2.1.1 De Novo Micro‐Thrombi

The composition of micro‐thrombi is highly dependent on the underlying etiology. *De novo* micro‐thrombi formed in the microcirculation are common in a wide variety of pathologies, including CSVD and secondary injury after traumatic brain injury (TBI). Despite their differences, both pathologies involve endothelial dysfunction leading to a pro‐thrombotic endothelial phenotype. In CSVD [19, 20, 21, 24], endothelial activation and inflammation caused by chronic vessel wall injury result in (among others) the release of von Willebrand Factor (vWF), tissue factor (TF), P‐selectin, and platelet activator with subsequent micro‐thrombi formation [19, 20, 21, 22]. TBI represents an acute mechanical insult to the brain, leading to secondary injury that includes BBB breakdown and leakage of brain‐derived extracellular vesicles into the circulation. This process activates systemic inflammation and coagulopathy. Beyond chronic and acute insults, *de novo* micro‐thrombi may also form in response to infectious disease, as exemplified by COVID‐19. SARS‐CoV‐2–induced endothelial activation results in a cytokine storm, neutrophil extracellular trap (NET) release, and impaired fibrinolysis—all of which contribute to intravascular thrombosis [25, 26].

#### 2.1.2 Migrating Micro‐Thrombi

In addition to de novo formation, micro‐thrombi may also originate elsewhere and lodge more distally. Two major sources of embolic micro‐thrombi are atherosclerotic plaque rupture and AF. In plaque rupture, mechanical disruption of a vulnerable atherosclerotic lesion—typically in the carotid artery or aortic arch—exposes highly thrombogenic material such as tissue factor, collagen, and lipids [27]. This triggers platelet activation and thrombin generation, resulting in a fibrin‐ and platelet‐rich thrombus [28]. Parts of this thrombus may fragment and enter the bloodstream, leading to distal ME [29]. In AF, blood stasis within the left atrial appendage promotes coagulation and thrombus formation. Upon distal lodging, microinfarcts can develop that are often silent but associated with cognitive impairment and increased stroke risk. While both conditions generate embolic micro‐thrombi, thrombi resulting from AF are typically older, more organized, and richer in fibrin, whereas those derived from plaque rupture are often younger, heterogeneous, and may contain cholesterol crystals or inflammatory cells [27, 30, 31, 32, 33]. Quantitative data on the number of thrombi and their sizes are lacking, but it seems reasonable to assume that the smallest thrombi occur by far the most frequently.

#### 2.1.3 Iatrogenic Micro‐Thrombi

Iatrogenic micro‐thrombi can arise as a consequence of neurovascular interventions, particularly during endovascular treatment like mechanical thrombectomy for large vessel occlusion [34, 35]. While mechanical thrombectomy has revolutionized acute ischemic stroke treatment, the procedure itself can inadvertently generate distal ME through fragmentation of the primary thrombus or dislodgement of endothelial or atherosclerotic debris [36, 37]. In larger thrombus fragments or large numbers of microscopic fragments can cause new infarcts following successful recanalization, as can be detected on diffusion‐weighted imaging [38, 39].

Iatrogenic micro‐thrombi may further result from catheter manipulation, contrast injection, or air embolism during diagnostic or therapeutic angiography [40, 41, 42]. To date, there are no direct histological studies characterizing the composition of iatrogenic micro‐thrombi formed during or after mechanical thrombectomy or other interventions. However, indirect evidence from retrieved thrombi suggests that fragments likely consist of fibrin‐ and platelet‐rich material [43, 44, 45].

### 2.2 Erythrocytes

Red blood cells (RBCs) normally pass the capillary bed by virtue of their flexibility and lack of adhesion proteins at the surface. Experimental stiffening of RBCs by oxidative stress causes their stalling in the microcirculation of the brain and other organs [46]. RBC stiffening occurs in sickle cell disease, contributing to sickle cell crises where the perfusion of the brain and other vital organs is compromised by micro‐occlusions. The process is far more complex than a mere ‘blockage of the capillary sieve’ by RBCs that are too stiff to pass through [47]. Rather, increased cell adhesion in the post‐capillary venules, endothelial inflammation, and expression of adhesion molecules are involved, leading to recruitment of more RBCs, activated leukocytes [48], and platelets, and finally a full micro‐occlusion [49]. Based on in vitro approaches and numerical analyses, Caruso et al. [47] showed that local forces on the endothelial cells are strongly affected by the occlusion, contributing to the endothelial inflammation.

Cerebral malaria involves RBC surface expression of antigens such as Plasmodium falciparum erythrocyte membrane protein 1 that bind to a variety of endothelial receptors and adherence of the RBC to the capillary and venular endothelium. This induces a further cascade of obstructive events as well as prothrombotic and inflammatory responses and damage to the BBB [50]. RBC stiffening and aggregation occur in diabetes [50], and possibly more generally in cardiovascular diseases [51, 52]. Mohaissen et al. [53] showed mutual aggravation between RBC stiffness and endothelial dysfunction in a mouse model. However, to the best of our knowledge, quantitative data on stalling by stiffened RBC or clusters of RBC in the brain are lacking.

### 2.3 Fat Particles

Since cerebral fat embolism (CFE) is a rare clinical condition, data are scarce, specific risk factors are unclear, and the pathophysiological mechanisms involved are still poorly elucidated [54, 55, 56, 57, 58, 59]. Moreover, no universal diagnostic criteria are defined [54, 55, 56, 57, 58, 59]. CFE develops through complex processes that are historically explained by two key theories: The “mechanical theory” describes the occurrence of fat emboli as a result of injury to adipose tissue in bone marrow, often following trauma or orthopedic surgery. Due to the occurrence of increased intramedullary pressure, fat particles enter the blood circulation. Within this theory, for cerebral involvement, fat globules must be small (< 10 μm) and numerous, as larger particles are filtered out by the lungs unless there is a right‐to‐left shunt (e.g., patent foramen ovale or intrapulmonary shunt), which allows access to the brain [57, 59, 60, 61]. The second “(bio)chemical theory” describes the massive release of plasma mediators (hormones), stimulating lipolysis in tissue after trauma, surgery, or certain nontraumatic diseases. This process causes the release of free fatty acids and other metabolites (prostaglandins, leukotrienes) into the circulation, causing local inflammation in the microvasculature and the formation of fat droplets [62, 63]. Neurological injury due to CFE results not only from physical vessel blockage but also from the toxic effects of these fatty acids.

CFE lacks a widely accepted diagnostic definition, and diagnosis relies heavily on clinical suspicion due to the absence of validated criteria [55, 56, 57, 58]. The neurological symptoms of CFE—ranging from headache and confusion to seizures and coma—are more variable and nonspecific than the respiratory symptoms seen in fat embolism syndrome, making diagnosis challenging. MRI is the most informative diagnostic tool, revealing multiple characteristics described across acute, subacute, and late stages, reflecting the dynamic pathological changes of CFE. These include white matter damage (atrophy or demyelination), edema (cytotoxic or vasogenic), and petechial hemorrhage [54, 64, 65, 66, 67, 68].

### 2.4 Gas Emboli

Gas emboli, also known as cerebral arterial gas embolism (CAGE), can block cerebral micro‐vessels with serious, potentially fatal consequences. CAGE can occur as a result of pulmonary barotrauma related to diving or during certain invasive medical procedures such as cardiac surgery, lung surgery, and endovascular procedures [69, 70]. A recent study found that 16% of patients who underwent mechanical thrombectomy for acute ischemic stroke had linear air bubbles visible on post‐procedure cerebral CT scans [71]. These larger, linear air emboli—distinct from smaller, spherical bubbles—were associated with worse short‐ and long‐term clinical outcomes compared to patients without such findings.

The pathophysiology of CAGE and bubble reabsorption and clearance rate depends on the size, location within the cerebral circulation, blood flow, gas composition, and treatment measures [72]. Gas emboli generally dissolve within minutes to a few hours, depending on the bubble's gas‐exchange area [73]. This results in a gradual decrease in size, to eventually be flushed out via the venous circulation [74]. Larger, elongated bubbles are more likely to become lodged, causing prolonged occlusion (up to several hours) and primary ischemic injury in the affected brain areas [75]. Smaller, spherical bubbles may pass through the vascular bed more quickly or be more rapidly reabsorbed [76]. Adhesion between bubbles and vessel walls may involve interactions between endothelial surface elements and plasma proteins adsorbed on the bubble [77]. Even brief contact with these gas emboli can damage the vascular endothelium by disrupting the glycocalyx and irritating the endothelial cells [77]. This endothelial injury triggers activation and inflammation, disrupts the BBB, and promotes cerebral edema and pro‐thrombotic processes, potentially leading to secondary ischemic injury even after the bubbles have cleared [78, 79].

### 2.5 Leukocytes

Leukocyte entrapment is a recognized phenomenon that can contribute to microvascular occlusion and embolization [80]. Cerebral blood flow (CBF) reductions or even stalling due to leukocyte entrapment have been linked both as cause and consequence to chronic (vascular) diseases, including hypercholesterolemia [80] and Alzheimer's Disease [80, 81], as well as to ischemic stroke and other acute insults [82, 83, 84].

In acute events, leukocytes are rapidly recruited into the cerebral microcirculation (hours). In this setting, neutrophils are the primary leukocytes adhering to the vessel wall (via specific adhesion molecules on the endothelial cells, e.g., ICAM‐1, VCAM‐1) [84, 85], whereafter (> 24 h) other leukocyte populations become more prominent in these adhesive interactions [86]. The accumulation of rolling and adherent leukocytes is accompanied by platelet recruitment. Activated neutrophils may aggregate with these platelets and produce Neutrophil Extracellular Traps (NETs) [87], again increasing cellular accumulation by forming a scaffold [26, 88, 89]. This eventually will physically obstruct the microvessel and may amplify the reduced or stalled blood flow in already affected tissue.

Leukocyte entrapment mechanisms involve both adhesive interactions and physical properties of leukocytes. Inflammatory mediators upregulate endothelial adhesion molecules, promoting leukocyte binding and subsequent sequestration within capillaries. Additionally, changes in leukocyte stiffness—modulated by hormones or cytokines—can influence margination (adherence to vessel walls) and demargination (release into circulation), potentially affecting the likelihood of entrapment [90]. Stiffer leukocytes may be more prone to becoming lodged in narrow vessels, especially in inflamed or injured brain tissue where endothelial activation is heightened.

### 2.6 Microplastics and Other Extrinsic Particles

Plastic nano‐particles are widely detected in the environment and are capable of entering the human body through the lungs, gastrointestinal tract [91] or skin and passing the BBB [92], possibly providing a health risk due to their toxicity. Of relevance for the current review is whether such particles can be large enough to become trapped in the microcirculation. Data on numbers and size distribution of plastic and other extrinsic particles in the blood is very sparse. Leslie et al. [93] reported detectable plastic particles of at least 0.7 μm in diameter in the blood, totaling 7.1 μg/mL. Their methods do not allow for the determination of particle size distribution. Other studies that did include particle size distribution showed that particles ranging in size from 1.4 to 57 μm can be detected in human whole blood [94], placenta [94], urine [95] and atheromatous plaques from carotid arteries [7, 96]. These extrinsic particles are sufficiently large to cause embolization [97], and there is no literature indicating that microplastics can dissolve intravascularly. It is therefore expected that trapped plastic particles may contribute to prolonged blood flow disturbances prior to clearance.

Micro‐ and nano‐plastics induce endothelial dysfunction in vitro by causing upregulation of adhesion molecules VCAM‐1 and ICAM‐1. In vivo, this would lead to enhanced recruitment and adhesion of leukocytes, oxidative stress, and pro‐inflammatory cytokine expression (IL‐1β and TNF‐α) [98, 99, 100, 101, 102, 103]. Additionally, microplastics can enhance thrombus formation by absorption of plasma proteins such as coagulation factor XII and plasminogen activator inhibitor‐1 [104, 105], underscoring the potential importance of micro‐plastics in the development of thrombosis.

## 3 Acute Consequences of Microvascular Embolization

**FIGURE 3:**
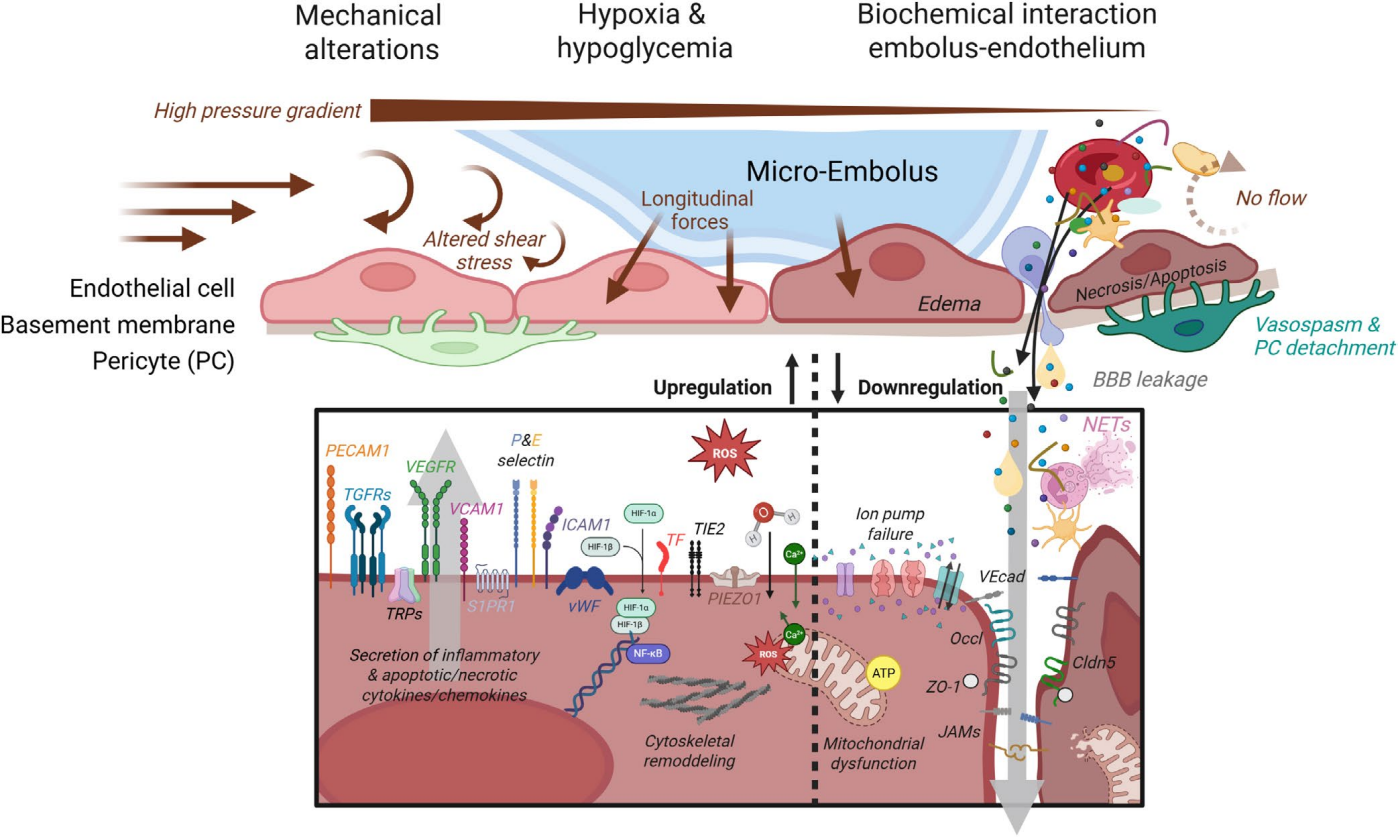
Brief summary of the biochemical signaling pathways upon embolization at the apical side of the endothelial cells. Pathways are activated due to alteration in mechanical forces and high pressure gradients, hypoxia and hypoglycemia and due to direct embolus‐endothelial interaction. ATP, adenosine triphosphate; Ca^2+^, calcium; Cldn5, Claudin‐5; ET‐1, Endothelin‐1; HiF1α, Hypoxia‐inducible factor 1 α; ICAM‐1, Intercellular adhesion molecule 1; JAMs, junctional adhesion molecules; Occl, Occludin; PECAM‐1, platelet and endothelial cell adhesion molecule 1; PIEZO‐1, large mechanosensitive ion channel protein; ROS, reactive oxygen species; S1PR1, sphingosine‐1‐phosphate receptor 1; TF, tissue factor; TGFRs, Transforming Growth Factor Receptors; TIE‐2, tyrosine kinase receptor (also known as TEK); TRPs, mechanosensitive transient receptor potential ion channels; VCAM‐1, vascular cell adhesion molecule‐1; VEcad, vascular endothelial cadherin; VEGFR2, vascular endothelial growth factor receptor 2; vWF, von Willebrand Factor; ZO‐1, zonula occludens 1. *Created in BioRender* [106].

The extensive cascade of pathological processes activated upon ME lodging starts with flow impairment (Figure 2). Additional processes might be initiated by the direct physical forces, hypoxia and hypoglycemia, and biological interaction with the endothelial cells. A brief overview can be found in Figure 3. The biological interactions may be extrapolated from in vitro and in vivo experiments, as indicated above for microplastics. There is a wealth of data here, which we do not discuss but which is extensively reviewed elsewhere [107, 108, 109, 110, 111, 112, 113, 114]. Mechanical interactions are more difficult to extrapolate from in vitro data. However, it is quite conceivable that a lodged ME exerts a large shear force on the endothelial cells. This is because wall shear stress is proportional to the pressure gradient. This gradient is very steep, as upstream pressure is increased and downstream pressure is decreased when a ME blocks flow. While initial computational approaches support this (data not shown), a more thorough mechanical analysis is needed to substantiate this point.

### 3.1 Flow Disruption

Penetrating arterioles are particularly vulnerable to flow impairment due to the lack of collateral pathways [115]. Occlusions in single penetrating arterioles (5–25 μm) or venules (20–100 μm) were shown to cause columns of tissue infarction with diameters around 500 μm and specific cognitive deficits [116]. Obstructions of larger vessels in the more proximal pial circulation in this model have fewer consequences due to the arcading nature of this part of the brain circulation [117]. A single ME lodging in the more distal bed of arterioles and capillaries branching off the penetrators is expected to have less acute effects because of the limited downstream tissue volume, diffusion of oxygen from other locations, and the alternative perfusion pathways present in the capillary plexus. Clearly, if sufficient numbers of ME lodge, perfusion and oxygenation would become impaired (Figure 1).

The consequences of micro‐embolization are a complex function of the number and size distribution of the particles. Georgakopoulou et al. injected polystyrene microparticles (MP) of 15–50 μm in diameter in the rat carotid artery and quantified the effect on ischemia, hypoxia, and infarction [118]. We fitted an allometric relation to their data and found the total ischemic volume to be proportional to the number of microparticles and the third power of the particle diameter. Individual particles of 15 or 25 μm impair capillary perfusion but do not cause infarction, in contrast to 50 μm particles. However, occasional infarcts are found in areas where multiple small MP lodge [118]. A further quantitative spatial analysis of tissue damage in this model revealed that a mixture of 15–50 μm MP causes numerous hypoxic regions after 24 h, whose volume and distance from the trapped MP were highly stochastic. MP trapping increased the risk for hypoxia in regions up to 1.5 mm away from the MP, and the risk for hypoxia was strongly amplified by the trapping of multiple MPs in the vicinity [119].

The above mentioned details all originate from rodent experiments. Data from humans is lacking. However, Xue et al. [17] simulated lodging of 25 μm ME in penetrating arterioles in a previously validated branching model for the human cortical columns. These simulations predict hypoxic regions downstream of the occlusion site that are comparable to experimental data on rats reported in the same study [17].

### 3.2 Local Consequences of Flow Disruption

Interruption of local oxygen and nutrient delivery leads to sudden cellular metabolic disruption, ATP depletion, and impaired ion pump function, disturbing calcium, sodium, and potassium gradients (Figure 3). This results in excitotoxicity, cytotoxic edema, and oxidative stress, including the production of cytotoxic reactive oxygen species (ROS) [105, 120, 121]. The disrupted calcium homeostasis also provokes dysregulated constriction, increasing microvascular resistance and worsening hypoxia [122].

The sequential endothelial dysfunction, driven by lower cAMP, increases vascular permeability and BBB leakage. The endothelium under stress also activates platelets via integrin‐mediated endothelial interaction [123], which further consolidates the embolus with persistent ischemia as a result.

Ischemic stress also triggers transcriptional reprogramming—suppressing oxygen‐sensing enzymes and activating hypoxia and inflammation pathways mediated by, among others, HIF‐1α and NF‐κB [124]. These changes recruit innate and adaptive immune responses [125, 126], for example via pattern recognition receptors like TLR4 [127]. The inflammatory reaction is controlled by the discharge of ROS, chemokines, and cytokines, which stimulate endothelial adhesion molecules, natural antibodies, and the complement system.

The combination of energy failure, ion imbalance, oxidative stress, and immune activation precipitates various forms of programmed cell death: apoptosis, autophagy‐associated death, necroptosis, and necrosis, each marked by distinct morphological changes [128, 129].

The excessive inflammation during the first days after occlusion forms a detrimental loop of action linked with BBB breakdown and neuronal injury, eventually leading to a worsened outcome [130].

## 4 Long‐Term Consequences of Microvascular Embolization

Acute experiments on rodents have shown that location, size, and number of microvascular occlusions determine the risk for infarction and, therefore, long‐term consequences [14, 118, 119, 131, 132]. These experiments are primarily relevant for cases where acute ‘showers of ME’ occur, such as in thoracic surgery or mechanical thrombectomy [34, 35, 36, 37, 40, 41, 42]. MEs in other cases, including AF [27, 30, 31, 32, 33] or the occurrence of microplastics [7, 33, 98, 99], become trapped at a slow but continuous rate, allowing the cerebral circulation to restore perfusion before the next event occurs and so avoiding infarction. These dynamics are therefore a critical determinant of long‐term consequences (Figure 1).

### 4.1 Vascular Reserve

While single ME may only cause reversible damage, both acute ‘ME showers’ and continuous trapping without adequate removal would be expected to lead to local perfusion deficits and, at a more global level, to impaired vascular reserve.

Vascular reserve refers to the capacity of blood vessels to increase blood flow above the resting level when tissue demand rises—for instance, during exercise or stress. It reflects how well the vasculature can adapt to increased metabolic needs. A healthy vascular reserve would allow at least a doubling of blood flow during increased demand [133]. The brain's vascular reserve can be measured by a vasodilatory stimulus such as CO_2_ elevation or acetazolamide with imaging by PET, CT/MR perfusion, or transcranial Doppler [133].

Sickle cell anemia forms a clear case where reserve is reduced [134], limiting the metabolic rate [135], very likely as a consequence of the ME. Also in AF, reserve is impaired [136], notably in hypertensives [137]. In other cases, the involvement of ME is less clear. Thus, the impairment of vascular reserve in aging and diabetes [138, 139] could partly relate to micro‐thrombi generated as a consequence of endothelial cell dysfunction and coagulation activation. An intriguing other condition associated with both micro‐thrombosis and impaired vascular reserve is traumatic brain injury [139]. It should be noted that impaired vascular reserve is usually associated with vasoreactivity rather than micro‐occlusions [139, 140, 141]. More direct evidence is therefore needed for the involvement of ME in impaired vascular reserve.

### 4.2 Cognitive Decline and Vascular Dementia

Vascular dementia is caused by impaired blood flow to specific regions of the brain, leading to tissue damage and progressive cognitive decline [142, 143, 144]. Atherosclerosis and CSVD are disorders that are frequently associated with both vascular dementia and ME formation [145, 146, 147]. The lodging of ME in small cerebral vessels is a major contributor to cerebral hypoperfusion. Disruptions in blood flow may lead to the formation of small infarcts [148, 149]. These small, often asymptomatic ischemic lesions in the white and deep gray matter can significantly increase the risk of dementia [16, 150, 151, 152]. Additionally, transcranial Doppler studies have linked spontaneous cerebral ME to accelerated cognitive decline and the progression of vascular dementia. In rodent models, the lodging of ME in small cerebral vessels reduces cerebral blood flow, leading to hypoxia, infarcts, and brain tissue damage in rats [14, 17, 119]. Overall, these studies support the central hypothesis that ME‐induced ischemia plays a key role in the development and progression of vascular dementia.

## 5 Clearance of Micro‐Emboli

In order to establish life‐long perfusion, MEs need to be removed from the capillary bed. Below, we discuss the clearance of micro‐thrombi by thrombolysis as well as the transcapillary migration of emboli toward the brain interstitium (Figure 2). In addition, microvascular patency could possibly be re‐established by rarefaction of the blocked capillary [153] and formation of new capillaries by angiogenesis. Angiogenesis in the brain is reviewed elsewhere [154]. Yet, to the best of our knowledge, angiogenesis specifically in response to local emboli has not been studied, and this possibility is not further discussed here.

### 5.1 Local Thrombolysis

Fibrin‐containing clots in the brain are subjected to digestion by plasmin, which is generated by tissue‐type plasminogen activator (tPA) that is locally produced by the endothelial cells and other cells of the neurovascular unit [155, 156], notably under ischemic conditions [156]. tPA activity is tightly controlled, ensuring efficient local activity at the clot without undesirable systemic effects. Yet, tPA activity can be impaired in several conditions. These include local endothelial dysfunction and elevated Plasminogen Activator Inhibitor‐1 (PAI‐1) levels as seen in metabolic syndrome and chronic inflammation [156, 157, 158]. While the roles of tPA and PAI‐1 in large arterial and venous thrombosis have been studied extensively, very little quantitative data is available on the lysis efficiency of micro‐thrombi in the brain in either chronic prothrombotic states or acute events such as ischemic stroke. As discussed above, ischemic stroke may cause inflow and local development of micro‐thrombi that overload the thrombolytic processes. Injected micro‐thrombi in a mouse model were found to be resistant to thrombolysis after three hours [11]. This was shown to be related to endothelial protrusions covering the micro‐thrombus. In addition, interaction with leukocytes may also prevent local thrombolysis, as seen in major clots [159]. Preventing such interactions reduces capillary stalls in a stroke model [82]. Therefore, while thrombolysis no doubt is a key process for normal thrombus clearance, it is quite conceivable that in chronic prothrombotic states and acute ischemic events, micro‐thrombi remain trapped in the microcirculation. This concerns native thrombolysis as well as thrombolytic therapy that targets micro‐thrombi [160, 161].

### 5.2 Extravasation/Angiophagy

A ME that remains lodged in the cerebral vasculature after failed intravascular breakdown can eventually be removed via endothelial transmigration across the BBB, a process also known as angiophagy, which was first described by Grutzendler and colleagues in 2010 [11]. Studies utilizing rodent models have demonstrated that MEs of different compositions undergo angiophagy at varying rates, subsequently entering the brain's parenchyma [11, 12, 132]. For example, exogenously introduced fibrin MEs were cleared within six days, whereas cholesterol crystal emboli extravasated within three to eight days [11, 12]. Analysis of *postmortem* rodent tissue in a silent brain infarct model revealed that polystyrene MPs of varying sizes can also be extravasated, although this process may take up to 28 days [131, 132]. Despite the relatively slow nature of angiophagy compared to thrombolysis, ischemia and hypoxia caused by MP occlusion in rats are reversible: studies show that functional recovery can occur within 7 days after inducing silent brain infarcts by polystyrene MP, highlighting the existence of repair mechanisms in this time frame. Additionally, Chang et al. [162] demonstrated endothelial phagocytosis of oxidatively stressed RBCs in vitro, and in subsequent in vivo studies, observed that stiffened RBCs stall and extravasate through the BBB in mice, with most extravasation occurring within 24 h [162, 163].

The composition of the ME appears to be a key determinant of extravasation rate: biological emboli such as fibrin and RBCs are cleared more rapidly than synthetic polystyrene MPs. Fibrin emboli, being susceptible to endogenous fibrinolysis, can fragment and thus accelerate their extravasation [11]. In contrast, the smooth surface and lack of protein interactions in polystyrene MPs likely contribute to their slower clearance. Our experimental work with biodegradable particles of different compositions further supports that extravasation rates depend on MP material [131].

Regardless of the composition of the ME, minimal to no protein leakage through the BBB is observed during angiophagy (own preliminary data), although transient micro‐glia activation does occur [11, 12, 131]. Following endothelial transmigration, fibrin MEs and RBCs are efficiently phagocytosed by microglia, whereas cholesterol crystals are engulfed but not fully degraded [11, 46]. Polystyrene MPs larger than 10 μm are typically not phagocytosed by microglia and can persist in the cerebral parenchyma [131, 132]. Phagocytosis of RBCs in particular may lead to the accumulation of interstitial iron, potentially contributing to neuroinflammation and the development of neurodegenerative diseases [164]. Despite multiple studies [11, 46, 131, 132, 162, 163, 164], our understanding of the ultimate fate of MEs after angiophagy remains very limited.

### 5.3 Balance of Micro‐Embolic Events and Micro‐Embolus Resolution

Above, we have characterized ME (occluding vessels of say 5–50 μm in diameter) as events that may not necessarily lead to acute micro‐infarction but could impair vascular reserve and resilience, increasing the risk of developing vascular dementia. The various clearance processes counteract the build‐up of emboli in the brain, forming a delicate equilibrium (Figure 2). Effects of micro‐occlusions depend on the type of embolus, event rate, size, location, and colocalization of multiple ME, as well as on the acute or chronic nature (colocalization in time) of these events. Clearance may involve several processes, partly depending on the nature and size of the embolus. Both occlusion and clearing can be affected in cardiovascular morbidities. Endothelial dysfunction may lead to increased numbers of circulating micro‐thrombi from atherosclerotic plaques or from a more pro‐thrombotic state. At the same time, this dysfunction may impair clearance, as endothelial cells play key roles in clearing. The picture thus emerges that in healthy individuals, microvascular clearing mechanisms effectively deal with the low rate of microvascular embolisms, but that both an increase in the microvascular event rate and less effective clearing ultimately may cause an imbalance that renders the cerebral circulation more and more vulnerable, eventually contributing to vascular origins of dementia. There is a large paucity of data here, among others on the event and recovery rates and distribution of ME sizes. The evidence supporting the existence of a gradually shifting balance is, at best, circumstantial and limited to specific conditions. In this review, we focused on micro‐thrombi but wanted to include other emboli because of their possible relevance for this balance.

## 6 Conclusion

The cerebral microcirculation is continuously challenged by micro‐embolic events. Repair mechanisms are needed to maintain adequate local perfusion throughout life. Depending on the nature of the embolus, these mechanisms include fibrinolysis or other intravascular degradation processes but also extravasation, transporting the emboli to the brain interstitium where further degradation occurs. The balance between events and repair requires a healthy endothelium, and endothelial dysfunction could lead to progressive loss of capillaries and functional consequences such as vascular dementia. Finally, non‐degradable microplastics might end up in the brain via such extravasation, inducing neuro‐inflammation and degeneration. There is a substantial lack of quantitative and mechanistic data here, and further research is warranted.

## Conflicts of Interest

The authors declare no conflicts of interest.

## Data Availability

Data sharing not applicable to this article as no datasets were generated or analyzed during the current study.
